# Supplementary material for: COVID-19 and Pulmonary Angiogenesis: The Possible Role of Hypoxia and Hyperinflammation in the Overexpression of Proteins Involved in Alveolar Vascular Dysfunction
Source: Viruses. 2023 Mar 8;15(3):706. doi: 10.3390/v15030706 (PMC10057465; doi:10.3390/v15030706)
Supplement: Supplementary file 1 [file viruses-15-00706-s001.zip › viruses-2257431-supplementary.pdf]

Supplementary Table S1. Antibodies sources.

Antibody	*Type*	*Clone/Code*	*Dilution*	*Source*	*Species Reactivity*	*Links*
Anti-ICAM-1	*Monoclonal/ Mouse*	*MA5-11433*	*1:100*	*Thermo Fisher*	*Human*	*https://www.thermofisher.com/order/genome-database/generatePdf?productName=ICAM-1&assayType=PRANT&detailed=true&productId=MA5-11433*
Anti-ANGPT-2	*Monoclonal/ Rabbit*	*A11306*	*1:100*	*AbClonal*	*Human, mouse*	*https://abclonal.com/catalog-antibodies/Angiopoietin2RabbitmAb/A11306*
Anti- IL-6	*Monoclonal/ Mouse*	*Ab9324*	*1:400*	*Abcam*	*Human, Rat*	*https://www.abcam.com/il-6-antibody-12-2b11-2g10-ab9324.html*
Anti -IL-1β	*Polyclonal/ Rabbit*	*A16288*	*1:800*	*AbClonal*	*Human, Mouse*	*https://abclonal.com/catalog-antibodies/IL1RabbitpAb/A16288*
Anti-vWF	*Polyclonal/ Rabbit*	*A0082*	*1:600*	*Dako*	*Human*	*https://www.agilent.com/cs/library/packageinsert/public/SSA0082IVD-US_01.pdf.*
Anti-PAI-1	*Polyclonal/ Rabbit*	*E-AB-40127*	*1:400*	*ElabSc*	*Mouse, Rat*	*https://www.elabscience.com/p-serpine1_polyclonal_antibody-26308.html*
Anti- CTNNB-1	*Monoclonal/ Rabbit*	*CAT-5H10*	*1:200*	*Thermo Fisher*	*Human*	*https://www.thermofisher.com/antibody/product/beta-Catenin-Antibody-clone-CAT-5H10-Monoclonal/13-8400*
Anti- GJA-1	*Polyclonal/ Rabbit*	*Ab235282*	*1:400*	*Abcam*	*Human, Rat, Mouse*	*https://www.abcam.com/connexin-43--gja1-antibody-ab235282.html*
Anti-VEGF	*Monoclonal/ Mouse*	*A17877*	*1:400*	*AbClonal*	*Human, Mouse, Rat*	*https://abclonal.com/catalog-antibodies/VEGFMousemAb/A17877*
Anti-VEGFR-1	*Polyclonal/ Rabbit*	*E-AB-65963*	*1:200*	*ElabSc*	*Human, Mouse, Rat*	*https://www.elabscience.com/p-vegf_receptor_1_polyclonal_antibody-389215.html*
Anti-NF-kB	*Monoclonal/ Rabbit*	*E381*	*1:400*	*Abcam*	*Mouse, Rat, Human*	*https://www.abcam.com/nfkb-p105--p50-antibody-e381-ab32360.html*
Anti-TNF-α	*Monoclonal/ Mouse*	*52B83*	*1:50*	*StaCruz*	*Human, Rat, Mouse*	*https://www.scbt.com/pt/p/tnfalpha-antibody-52b83*
Anti-HIF-1α	*Monoclonal/ Rabbit*	*EP118*	*1:200*	*BioSB*	*Human, Rat*	*https://www.biosb.com/biosb-products/hif-1-alpha-antibody-rmab-ep118/*

Legend:
Anti-ICAM-1 (Intercellular Adhesion Molecule-1or CD54),
Anti-ANGPT-2 (Angiopoietin-2),
Anti- IL-6 (Interleukin-6),

Anti-IL-1β (Interleukin-1β),
Anti-vWF (von Willebrand Factor),
Anti-PAI-1 (Plasminogen Activator Inhibitor-1),
Anti-CTNNB-1 (β-Catenin-1),
Anti-GJA-1 (Gap Junction Alpha-1 or Connexin-43),
Anti-VEGF (Vascular Endothelial Growth Factor),
Anti-VEGFR-1 (VEGF Receptor-1),
Anti-NF-kB (Nuclear Factor Kappa-Light-Chain-Enhancer of Activated B Cells),
Anti-TNF-α (Tumor Necrosis Factor- α),
Anti-HIF-1α (Hypoxia-Inducible Factor-1α).

Supplemental Table S2. Comparison between COVID-19, H1N1 and CONTROL groups according to immunohistochemical findings.

Biomarkers	CONTROL %/HPF	COVID-19 %/HPF	H1N1 %/HPF
Anti-ICAM-1[1]	0.55 (0.24-2.29)	7.67 (2.02-23.34)	0.24 (0.06-1.54)
		*p<0.0001	*p<0.0001
Anti-ANGPT-2[1]	4.15 (0.58-6.32)	8.78 (2.28-26.7)	4.03 (1.30-25.5)
		*p=0.0055	*p=0.1001
Anti-IL-6[1]	3.94 (1.15-26.91)	2.7 (0.28-13.92)	2.95 (0.45-10.62)
		*p=0.6064	p=0.9849
Anti-IL-1β[1,a]	6.18 (0.82-16.93)	19.5 (6.1-39.86)	14.1 (2.64-24.6)
		*p=0.0002	*p=0.0221
Anti-vWF[1]	4.41 (1.07-13.95)	2.02 (0.68-25.77)	3.45 (0.14-11.9)
		#p=0.1716	
Anti-PAI-1[1]	24.53 (12.93-43.82)	14.88 (2.88-44.57)	16.28 (4.63-31.64)
		*p=0.0276	p=0.6566
Anti- CTNNB-1[1]	1.51 (0.43-3.28)	17.83 (7.12-29.41)	1.91 (0.18-6.85)
		*p<0.0001	*p<0.0001
Anti-GJA-1[1]	9.5 (0.15-21.7)	16.4 (3.18-33.58)	5.52 (1.9-21.1)
		*p=0.019	*p=0.005
Anti-VEGF[1]	8.65 (4.08-11.76)	23.67 (2.70-44.86)	42.8 (26.7-56.9)
		*p=0.0024	*p=0.0009
Anti-VEGFR-1[1]	2.79 (0.12-10.21)	6.81 (1.79-20.16)	4.84 (0.46-19.2)
		*p=0.0032	*p=0.2766
Anti-NF-kB[1]	0.9 (0.11-4.85)	10.22 (1.61-16.1)	3.35 (0.37-6.81)
		*p<0.0001	*p=0.0002
Anti-TNF-α[1]	3.20 (0.35-10.12)	11.36 (2.2-25.99)	16.5 (8.78-25.99)
		*p=0.0006	*p=0.089
Anti-HIF-1α[1]	3.12 (0.23-8.28)	3.50 (0.80-21.33)	3.19 (0.89-16.0)
		#p=0.6446	

Legend: [1]Median (Min-Max); [a]n=15; * = p-values obtained were compared between COVID-19 vs. CONTROL group and COVID-19 vs. H1N1 group; # = p-values obtained were compared the three groups; The p-values were obtained using the non-parametric Kruskal-Wallis test, and when there was significant, the Mann-Whitney test was applied in the two-by-two comparison (p<0.05).

Supplemental Table S3. Comparison between patients in the COVID-19 group with or without type 2 diabetes mellitus as a chronic disease.

Diabetes Mellitus 2			
**Biomarkers**	**No (n=13)***	**Yes (n=11)***	***p*-value**
ICAM-1	7.38	9.54	0.691
ANGPT-2	10.48	5.84	0.733
IL-6	2.92	2.48	0.664
IL-1beta	18.15	19.50	0.622
PAI-1	13.27	15.27	0.776
vWF	1.44	2.55	**0.032**
VEGF	24.14	23.20	0.955
VEGFR-1	7.89	5.65	0.361
GJA-1	13.12	18.21	0.119
CTNNB	15.69	19.74	0.361
NF-kb	11.00	8.09	0.186
HIF-1a	3.32	2.76	0.733

* Median values of tissue expression of biomarkers.

Supplemental Table S4. Comparison between patients in the COVID-19 group with or without chronic use of platelet antiaggregant.

Chronic use of platelet antiaggregant			
**Biomarkers**	**No (n=09)***	**Yes (n=15)***	***p*-value**
ICAM-1	7.38	9.54	0.189
ANGPT-2	4.51	10.48	0.144
IL-6	2.21	6.02	**0.004**
IL-1beta	19.07	18.15	0.928
PAI-1	13.27	15.26	0.531
vWF	1.44	2.11	0.270
VEGF	29.50	21.10	0.126
VEGFR-1	5.65	7.07	0.165
GJA-1	13.10	18.02	0.278
CTNNB	15.70	18.00	0.542
NF-kb	9.70	10.6	0.429
HIF-1a	4.00	3.13	0.953

* Median values of tissue expression of biomarkers.

Supplemental Table S5. Comparison between patients in the COVID-19 group with or without chronic use of anticoagulants.

	**Chronic use of anticoagulants**		
**Biomarkers**	**No (n=20)***	**Yes (n=04)***	***p*-value**
ICAM-1	7.59	11.71	0.241
ANGPT-2	8.78	8.23	0.911
IL-6	2.48	7.50	0.131
IL-1beta	18.61	16.30	0.757
PAI-1	13.88	22.46	0.135
vWF	1.49	2.02	0.462
VEGF	24.44	17.43	0.309
VEGFR-1	6.82	9.80	1.000
GJA-1	15.09	19.30	0.852
CTNNB	16.67	19.44	0.911
NF-kb	9.79	10.80	0.970
HIF-1a	3.20	3.86	0.682

* Median values of tissue expression of biomarkers.

Supplemental Table S6. Comparison between patients of the COVID-19 group that used or did not use corticosteroids during hospitalization.

	**Corticosteroids**		
**Biomarkers**	**No (n=07)***	**Yes (n=17)***	***p*-value**
ICAM-1	9.54	7.54	0.581
ANGPT-2	11.04	5.84	0.077
IL-6	6.97	2.48	**0.049**
IL-1beta	13.10	19.50	**0.018**
PAI-1	17.62	14.49	0.224
vWF	1.32	2.29	**0.042**
VEGF	14.43	26.56	**0.040**
VEGFR-1	10.05	5.65	0.137
GJA-1	13.72	18.04	0.310
CTNNB	14.37	18.69	0.415
NF-kb	13.43	9.70	0.053
HIF-1a	3.28	3.32	0.626

* Median values of tissue expression of biomarkers.

Supplemental Table S7. Comparison between patients in the COVID-19 group who had a PaO_2/FiO_2 ratio < 150.

	PaO_2/FiO_2 ratio < 150		
**Biomarkers**	**No (n=10)***	**Yes (n=13)***	***p*-value**
ICAM-1	6.68	7.79	0.446
ANGPT-2	12.06	4.61	0.101
IL-6	3.34	2.48	0.535
IL-1beta	16.07	19.50	0.215
PAI-1	13.88	16.55	0.927
vWF	1.78	1.93	0.738
VEGF	14.09	31.90	**0.008**
VEGFR-1	7.48	5.65	0.738
GJA-1	12.19	20.55	0.101
CTNNB	14.01	18.69	0.115
NF-kb	11.10	9.89	0.563
HIF-1a	4.14	2.67	0.115

* Median values of tissue expression of biomarkers.
